# The Effect of ω3 Fatty Acids Supplementation on Levels of PPARγ and UCP2 Genes Expression, Serum Level of UCP2 Protein, Metabolic Status, and Appetite in Elite male Athletes: Protocol for a Randomized Control Trial

**DOI:** 10.29337/ijsp.161

**Published:** 2021-08-19

**Authors:** Sara Moradi, Mohamadreza Alivand, Yaser KhajeBishak, Mohamad AsghariJafarabadi, Maedeh Alipour, Amirhosien faghfouri, Beitullah Alipour

**Affiliations:** 1 Student’s Research Committee, Department of Nutrition, Tabriz University of Medical Sciences, Tabriz, I.R., IR; 2 Department of Medical Genetics, Faculty of Medicine, Tabriz University of Medical Sciences, Tabriz, IR; 3 Department of Nutrition, Maragheh University of Medical Sciences, Maragheh, IR; 4 Department of Statistics and Epidemiology, Faculty of Health, Tabriz University of Medical Sciences, Tabriz, IR; 5 Medical Student, Faculty of Medicine, Tabriz University of Medical Sciences, Tabriz, IR

**Keywords:** Omega3 fatty acid, supplementation, PPARγ, UCP2, gene expression, body composition

## Abstract

Some genetic factors may influence body composition, such as PPARγ and UCP2. PPARγ plays an important role in body fat distribution. The objective of the present study is to determine the effects of omega3 fatty acids on the gene expression of PPARγ and UCP2, levels of blood lipid profile, fat mass, and fat-free mass, and appetite.

Elite male athlete volunteers of up to 36 subjects were invited to participate in this RCT. Following a public announcement, volunteers were recruited from gyms, teams, and sports medicine boards in Tabriz, Iran. Gene’s expression of PPARγ and UCP2, serum levels of blood lipid profile, fat mass, and fat-free mass was collected. Data collection time points include baseline in addition to 3 weeks follow up. The study was approved by the Ethics Committee of the Tabriz University Medical of Sciences (IR.TBZMED.REC.1398.782) in October 2019 and was registered with the Iranian Registry of Clinical Trials: 20190625044008N1 on December 19, 2019.

Recruitment began in July and concluded in December 2019. As of August 19, 2019, we have screened 373 volunteers. 36 were enrolled. Baseline measurements of participants were collected. After three-week of intervention, end study measurements of participants were collected. The results are expected to be released in 2021. Participants have a median age of 21.86 (±3.15). The finding of this study showed Results showed PPARγ mRNA levels, and UCP2 mRNA and protein levels increased in the omega3 group (p < 0.05), as did REE (p < 0.05). Also, differences in the sensation of hunger or satiety were significant (p < 0.05).

This study could result in the effects of omega-3 fatty acids on PPARγ, and UCP2 expressions, blood lipid profiles and body composition. In addition, the results of this trial can be used as baseline information for conducting further clinical and sport nutrition studies.

**Trial Registration:**

The trial was registered at the Iranian registry of the clinical trial website (*www.irct.ir*) as IRCT20190625044008N1 (*https://en.irct.ir/trial/43332*), registered at (19/12/2019).

**Highlights:**

- Omega3 fatty acids as a ligand of metabolic-related genes, have a role in energy expenditure.
- Omega3 supplements effect on PPARγ and UCP2 mRNA expression as regulators of energy metabolism
- Omega3 supplements increased REE.
- Omega-3 supplementation could change the changes in body composition.
- For athletes, omega-3 simultaneously decreased fat mass and increased fat-mass.
- HDL-C increased after short-term supplementation with omega-3.
- Increased intake of omega-3, caused increased intake of energy and protein.

Clinical laboratories and technical departments and/or involved in the research:

- Department of Medical Genetics, Faculty of Medicine, Tabriz University of Medical Sciences, Tabriz, Iran
- Department of Community Nutrition, Faculty of Nutrition, Tabriz University of Medical Sciences, Tabriz, Iran

## Introduction

Overweight is one of the leading causes of disease in adults [1]. Prevention and treatment of obesity and its comorbidities have received many interests in the last decades [2,3]. One of the main preventive strategies for reducing obesity is lifestyle modification [4,5]. Until now many techniques such as healthy eating and regular physical activity have been suggested for this purpose [6]. However, many factors impress the response of individuals to lifestyle changes [7,8]. For example, reduced energy intake and/or increased physical activity by changing the metabolism of energy could prevent obesity [9]. But the exact change is different in people. One of the reasons for this difference may be differences in gene expression. About 127 genes have been identified as metabolism-related genes and genome-wide association studies (GWAS) have validated approximately 50 obesity susceptibility genes [10,11,12], and genome-wide association studies (GWAS) revealed the robust association of the gene with adiposity, body weight, BMI or class of obesity, and percent body fat [13].

Usually, obesity is identified with increased fat mass (FM) and decreased fat-free mass (FFM) [14]. On the other hand, for many elite athletes, the ratio of FM and FFM is important, such as material arts. Many studies showed body composition, especially body fat percentage in athletes is less than non-athletes, and the amount of FM related to the metabolism of energy [15,16].

As athletes became more active, they used more energy, so they showed more expression of metabolism-related genes [17]. In addition to gene expression, biochemical and environmental elements affect the metabolism of energy while these factors interact with each other.

For example, fatty acid-binding protein-4 in complex with omega3 fatty acids activates peroxisome proliferator-activated receptor (PPARγ) and in turn, this factor regulates metabolism [18,19]. As noted in many studies, PPARγ is a transcription factor expressed in adipose tissue that determines the distribution of FM in the body [18,20] So, increasing the expression of genes related to exercise that boost the body’s metabolism can reduce the incidence of obesity, metabolic and cardiovascular diseases [21,22].

Omega3 fatty acids include alpha-linolenic acid, eicosapentaenoic acid (EPA) and docosahexaenoic acid (DHA). They are important constituents of phospholipids in cell membranes that play an important role in fluidity and membrane integrity [23,24]. In addition, omega3 fatty acids affect the transcriptional regulation of genes through various pathways, resulting in decreased blood lipids and body fat [25]. As omga3s are ligands of PPARγ, they can alter metabolic markers associated with energy metabolism, blood triglycerides, and body composition [26,27].

**Figure 1:**
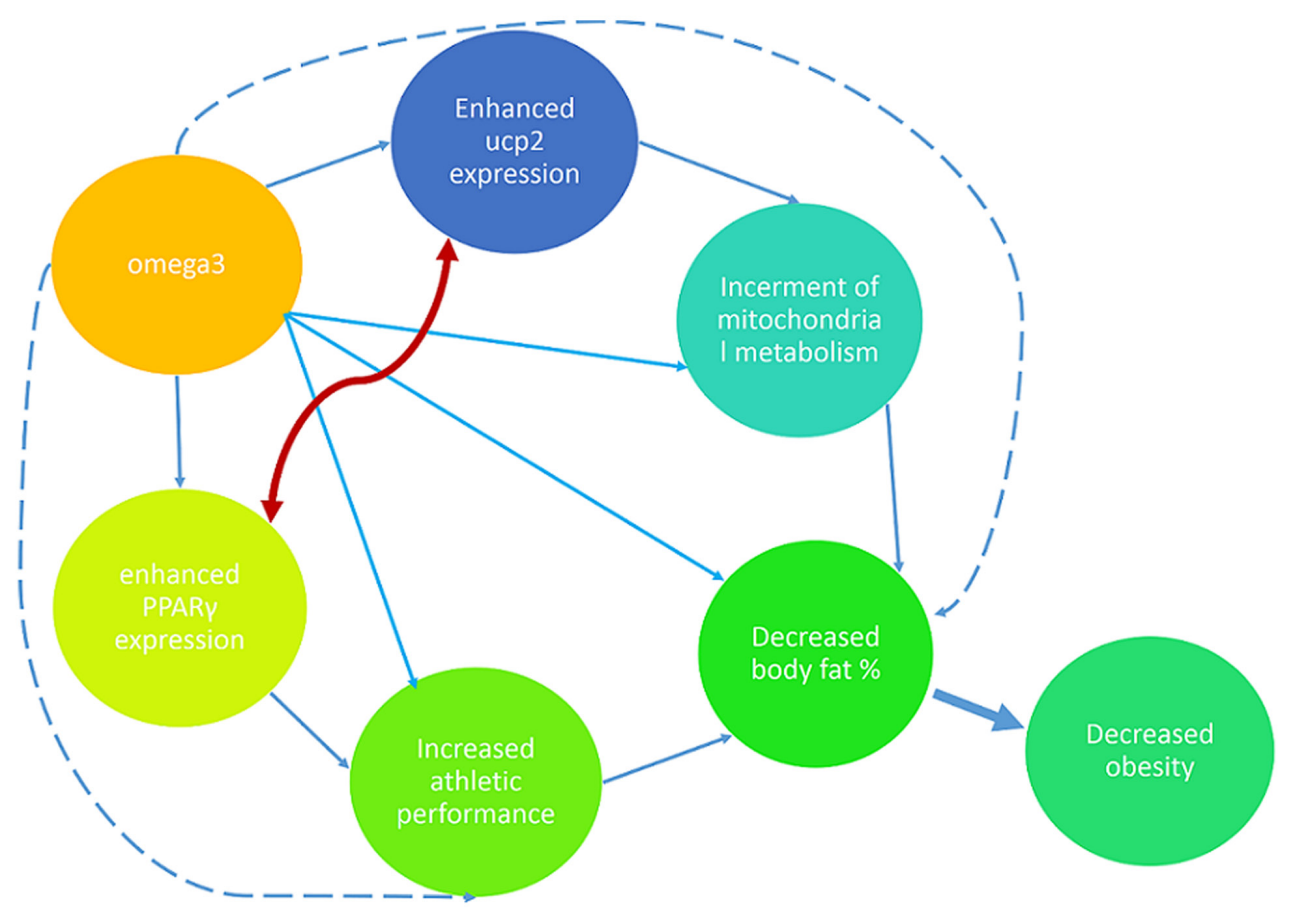
The hypothetic effect of omega3 supplementation on obesity.

Increased expression of PPARγ activates other genes related to energy metabolism, such as the Uncoupling protein2 (UCP2) [28]. Uncoupling proteins (UCPs) are located in the inner mitochondrial membrane and cause proton leakage from the space between the two membranes of the mitochondrial matrix. UCPs by hydrogen leakage reduce the electrochemical hydrogen gradient involved in ATP production [29]. Due to the role of UCP2 in energy metabolism, the lack of this protein disrupts mitochondrial function in patients with obesity. As well as, higher levels of this protein lead to the better oxidation of fatty acids [30]. Various studies have investigated lower UCP2 gene expression in metabolic and cardiovascular diseases [25,31]. On the other hand, higher levels of UCP2 protein lead to the better oxidation of fatty acids [32]. In addition to the role of omega3 supplementation on PPARγ, they increased UCPs gene expression in muscle cells and thus affect energy expenditure and lipid oxidation, however, the results are controversial. On the other hand, UCP2 gene expression up-regulates PPARγ expression [33]. The hypothetic effect of omega3 supplementation on obesity showed in ***Figure 1***. Although, omega-3 supplementation has psychological aspects that could affect the mood and tendency to physical activity, therefore, the effect of omega-3 on obesity can be studied from different [34,35].

From the past decades, studies showed the rate of fat and muscle mass in the body, directly associated with energy metabolism [36,37]. Also, several studies have examined the effects of This study aims to investigate the effect of omega 3 fatty acid supplementation on energy-related metabolism (PPARγ and UCP2) by considering the changes in body composition, blood lipids, appetite, and dietary intake.

### Study objectives

Primary objective: Omega3 fatty acid supplementation affects the body metabolism in the intervention and control group.

### Secondary objective

1. Omega3 fatty acid supplementation affects the level of expression of the UCP2 and serum level of the UCP2 protein.
2. Omega3 fatty acid supplementation affects the gene expression level of the PPARγ.
3. Omega3 fatty acid supplementation affects resting energy expenditure (REE).
4. Omega3 fatty acid supplementation affects body composition, lipid profile, appetite, dietary intake.

## Methods

### Study design

**Figure 2:**
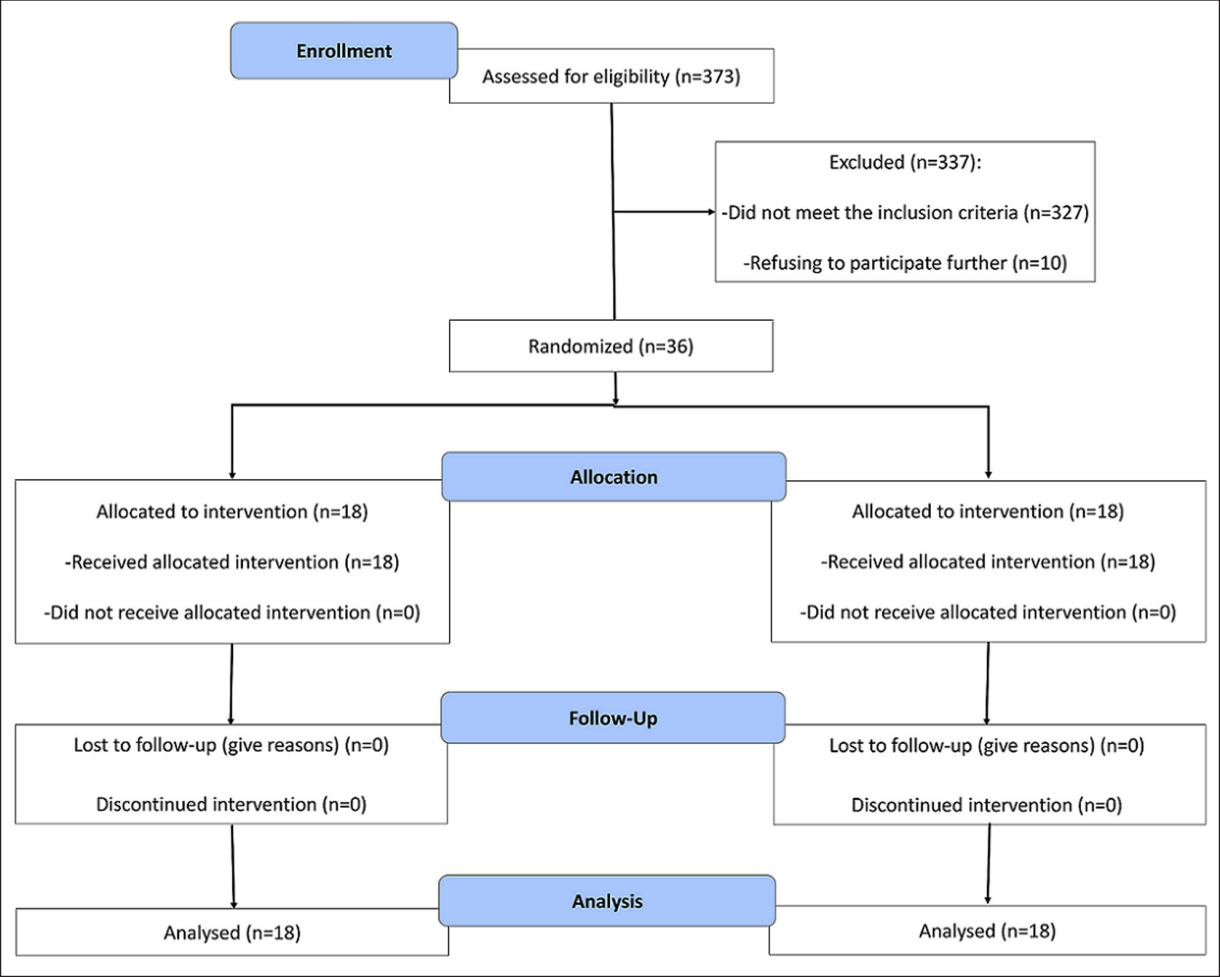
The diagram of the study design .

This was a randomized double-blind placebo-controlled trial involving 36 athlete men. The duration of the project was three weeks. Following a public announcement of the study, volunteers who willing to participate were recruited from public and private gyms, teams, stadiums, councils, and departments of sports, departments of physical education, and the sports medicine board in Tabriz, Iran. After being given a full explanation of the study procedures, participants who agreed to enroll in the study signed a statement of informed consent before the commencement of baseline data collection. The diagram of the study design is shown in ***Figure 2***. In addition, ***Table 1*** contains the Time-points for the study protocol.

**Table 1:** Time-points for the study protocol.


	STUDY PERIOD			

	ENROLMENT	ALLOCATION	POST-ALLOCATION	CLOSE-OUT

TIMEPOINT	Before enrollment	0	Week 0	Week 3

ENROLMENT:				

Eligibility screen	X			

Informed consent	X			

Allocation		X		

INTERVENTIONS:				

[Intervention omega3]			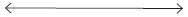	

[placebo]			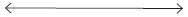	

ASSESSMENTS:				

Physical activity level		X	X	X

VAS			X	X

Dietary intake			X	X

Body composition		X	X	X

Lipid profile			X	X

UCP2 gene expressions			X	X

PPARγ gene expressions			X	X


### Inclusion/exclusion criteria

The inclusion criteria were: 1) athlete volunteers who were ranked nationally or players of a professional sports league (football, volleyball, swimming, etc.); 2) age range of 20 to 30 years; 3) BMI between 18.5 to 25 kg/m^2^; 4) avoidance of any dietary supplements, vitamins, minerals, and protein powders at least six months before and throughout the intervention; 5) Not having a history of coagulopathy blood disease, liver damage, kidney disease, pancreatitis, inflammatory diseases, diabetes, cancer, thyroid disorders, and heart disease; 6) not smoking. The exclusion criteria were: 1) allergic response to the omega3; 2) unwillingness for cooperation; 3) any major change in diet, duration, level, or type of physical activity and regular lifestyle.

### Enrollment, Randomization, Blinding, and Fallow-up

Participants who met the eligibility criteria were randomly assigned to the omega3 (n = 18) and placebo (n = 18) groups. For randomization, a blinded colleague who was not involved in any of the study stages randomly divided the participants into the intervention and placebo groups (1:1) by using RAS (Random Allocation Software). Omega 3 or placebo containers with identical labeling and were similar in terms of color, shape, and size. Gelatin capsule supplements and placebo were stored at room temperature. The adequate intake of omega3 for men between the ages of 19–50 years is 1600 mg per day. In similar previous studies, the dose of omega3 supplementation ranged from 200 mg to 6 g per day, and by considering the low amount of omega3 in the Iranian diet, an effective dose at 2 g per day was intended for this study [51–54].

Participants were stratified into two groups:

1. The Omega 3 group receiving supplements of two Omega 3 soft gel capsules per day (Zahravi Pharmaceutical Co, Tabriz, Iran, consists of 240 mg of DHA, 360 mg EPA).
2. The placebo group receiving placebo two soft gel capsules per day, each capsule containing one g of edible paraffin oil (provided by Zahravi Pharmaceutical, Co., Tabriz, Iran).

Participants were asked to return bottles of supplement and the compliance of participants was evaluated by counting the number of unconsumed capsules at the end of the intervention. None of the participants who completed the trial had compliance less than 90% (38 out of 42); therefore, no participants were excluded for inadequate compliance. Participants were advised to maintain their regular diet and level of physical activity during the study. Participants were contacted weekly to track any problems or adverse events, reminded to take their supplements and to evaluate whether diet or physical activity had changed. None of the participants were excluded because of substantial changes in diet or physical activity. Adverse events were also tracked for a week after the end of the intervention.

### Data Collection

For general information questionnaire, the participants were asked about age, education, job, smoking, alcohol consumption, medical history includes a history of diseases such as diabetes mellitus, renal and liver disorder, myocardial infarction, stroke, asthma, allergy, cancer, immune disease, history of drug consumption, and use of vitamin/minerals supplements.

Other questionnaires were completed before and after the study. For nutritional assessment food frequency questionnaire (FFQ) was validated for the Iranian population [38], and the 24-hour dietary recall questionnaire at the beginning and the end of the study consist of two typical days and a weekend day was taken. The dietary records are based on estimated values in household measurement. Data on food intake was analyzed by Nutritionist IV software (First Databank, San Bruno, CA, USA).

The patient’s physical activity level was estimated by the global physical activity questionnaire (GPAQ), through a face-to-face interview [39]. This questionnaire is a simple validated screening tool for ranking the physical activity of adults by focusing on current general activities [40]. Participants were advised to report time spent on moderate or vigorous intensity, walking, and sitting down during the week before the test. This questionnaire consists of questions asking about how much time the respondent has spent doing each of the intensity-varied activities during the last week; finally, a total metabolic equivalent (MET-minutes/week) score was calculated, and the participants were categorized as having a high, moderate, or low level of activity, according to the manual.

Data were processed according to guidelines for analysis of the GPAQ and total metabolic equivalents score (MET-minutes/week) was calculated, with participants categorized as high (≥3000 METs), moderate and low (<3000 METs) levels of activity. For assessing appetite, a 10 cm visual analog scale (VAS) questionnaire (with six items: hunger, satisfaction, desire to eat, desire to eat sweet, desire to eat salty, desire to eat fatty) was completed in the morning after fasting and after giving blood samples. The validity and reliability of this questionnaire were previously reported [41].

### Anthropometric Measurement

Anthropometric parameters including weight and height were measured. The weight of the participants measured with minimal clothing and without shoes by digital scale to the nearest 0.05 kg. Height measured using a wall stadiometer in standing position without shoes with a precision of 0.5 cm (Seca, Germany) [37]. Wrist circumference measured using a tape meter to the nearest 0.1 cm. BMI was calculated as weight (kg) divided by height squared (m2). Body composition evaluated using Tanita MC-780 S MA (Amsterdam, the Netherlands). RMR was measured by indirect calorimetry using Fitmate Pro (Rome, Italy). The measurements were performed by a trained nutritionist. Besides, blood pressure was measured in a comfortable sitting position in the left arm after at least five minutes resting, using an aneroid sphygmomanometer and stethoscope. It was measured on two occasions and the mean of the two was taken as the individual’s blood pressure.

### Sample blood collection, and ELISA

5 ml of whole blood was collected from all participants after 10–12 hours of overnight fasting. 1 ml was transferred to a sterile microtube without any anticoagulant, centrifuged at 3000 RPM for 5 minutes, and the separated serum stored at -70°C until UCP2 was measured. The enzyme-linked immunosorbent assay (ELISA) method was applied to measure serum UCP2 protein by commercial kits (Shanghai Crystal Day Biotech Co., LTD, China) (Intra-assay Precision (Precision within an assay): CV% < 8%; Inter-assay Precision (Precision between assays): CV% < 10%).

### PBMC separation, and gene expression

For measurement of gene expression, the remaining 4 ml of whole blood was used for the isolation of peripheral blood mononuclear cells (PBMCs) immediately after collection in tubes containing EDTA with anticoagulant (Vacutainer K2E). First, PBMCs were isolated by Ficoll Hypaque density-gradient centrifugation (Miltenyi Biotech GmbH, Bergisch Gladbach, Germany) and used immediately after collection. Total RNA purification was done by using the Ambion trizol reagent (Thermo Fisher Scientific), according to the manufacturer’s protocol. The quantity and quality of the RNA were assessed by using NanoDrop Spectrophotometry (NanoDrop OneC; Thermo Fisher Scientific). Then, complementary DNA (cDNA) synthesis was done according to the manufacturer’s protocol (ExcelRT One-Step RT-PCR Kit; smobio).

The integrity of the total RNA of the participants’ samples was assessed by gel electrophoresis (on a 1% agarose gel). For Real-time polymerase chain reaction, the PPARγ and UCP2 gene sequences were taken from the National Center for Biotechnology Information (NCBI) and Ensembl (*http://asia.ensembl.org/*) databases. The OLIGO7 Software (Molecular Biology Insights, Inc., Cascade, CO) was used for designing the primer pairs PPARγ and UCP2 of mRNA sequence. ***Table 1*** showed the sequences of PPARγ, UCP2, and glyceraldehyde-3-phosphate dehydrogenase (GAPDH) primers for the polymerase chain reaction. The level of PPARγ and UCP2 mRNA was examined using SYBR Green Master Mix (RealQ Plus 2x Master Mix Green, ampliqon, Denmark). The primer sequences for the human genes of PPARγ, UCP2, and GAPDH (as a housekeeping gene) were evaluated, and the data normalized to GAPDH mRNA expression by using the ΔΔCT comparative method. The fold change of the PPARγ and UCP2 mRNA were calculated by using the REST Software as the relative expression of post-intervention/placebo [62].

### Data Management and Analysis

STATA 15 software (StataCorp, College Station, Texas 77845 USA) was used to import and data analyze. All main analyses were to be based on intent to treat. Kolmogorov-Smirnov and Levene tests were used to check whether the data distribution is normal, respectively. If the distribution is normal, quantitative data were presented as mean and standard deviation and if abnormal, it was presented as 25–75 percentiles. Qualitative data also be provided in abundance (percentage). To compare the effect of supplemental supplementation before and after intervention in each group, the Paired t-test was used if normal and the Wilcoxon Sign Rank test was used if it is abnormal. To compare intergroup changes, the Independent sample t-test was used if normal, and the Mann-Whitney U test was used if it is abnormal. The Analysis of Covariance (ANCOVA) test was used to determine the net effect of the intervention. In all analyses, P values less than 0.05 were considered significant.

### Sample Size

A total of 36 subjects (men, 20–30 years old) were recruited to participate in this RCT study. The data were collected in Tabriz University of Medical Sciences (department of medical genetics for laboratory analyses and department of community nutrition for anthropometric and BIA measurements).

The sample size was estimated by considering the expected differences (d) between the two studied groups for one of the outcomes (REE was used from a previous clinical trial [42]). We calculated the sample size as follows: Z1–α/2 = 1.96 α = 0.05 1–β =0.90 Z1–β = 1.282

n = \frac{{\left( {{{\left( {\left( {z1 - \frac{\alpha }{2}} \right)\left( {z1 - \beta } \right)} \right)}^2}{\rm{*}}{{\left( {SD1 + SD2} \right)}^2}} \right)}}{{{{\left( d \right)}^2}}}

According to the equation above, the sample size was calculated by nearly 14 in each group and we selected 18 in each group, including a possible 30% (4 out of 14) loss to follow-up and discontinued intervention, and for great accuracy.

### Outcome Measures

The primary outcome measure is the change in gene expressions from baseline to the final week in intervention and placebo groups.

Secondary outcomes are changes in the below items from baseline to the end of the intervention:

- Anthropometric measures
- Body composition
- Resting metabolic rate
- Blood pressure
- Appetite status
- Serum UCP2 protein level
- Blood lipid profile
- Dietary intake

### Ethical Considerations

All participants received written informed consent. After being given a full explanation of the study procedures, participants who agreed to enroll in the study signed a statement of informed consent before the commencement of baseline data collection. The study procedure and the informed consent form were approved by the ethics committee of the medical university of Tabriz (IR.TBZMED.REC.1398.782) in October 2019, and all procedures were performed by relevant guidelines.

## Results

Enrollment began in July 2019 and the study concluded in August-September 2019. Of the 374 screened participants, 36 were enrolled. The questionnaires were administered during enrollment. For each participant, anthropometric parameters were measured, blood samples were collected at baseline and end of the study. FFQ, 24- hour food recall, VAS, and GPAQ were completed before and after the intervention from July to September 2019. Follow-up by phone conducted at the end of each week. A total of three-week supplementation were conducted. Protein level messaged in December 2019. The initial process of gene expression was conducted in January 2020, and the final assessments were in February 2020. The results are expected to be released in 2021. The finding of this study showed Results showed PPARγ mRNA levels, and UCP2 mRNA and protein levels increased in the omega3 group (p < 0.05), as did REE (p < 0.05). Also, differences in the sensation of hunger or satiety were significant (p < 0.05).

## Discussion

The main findings of this study were omega 3 supplementations could alter the expression level of PPARγ and UCP2. According to the evidence, polyunsaturated fatty acids with similar mechanisms have an important role in determining the severity of obesity, increasing fat-free mass, and reducing fat mass. Dietary supplementation with omega3 fatty acids may have clinically useful effects on decreasing the percentage of fat in the body. Although the mechanisms of omega3 effect on body fat are not recognized, the role of genetic polymorphisms and gene expressions have been approved in animal and human studies. For example, overexpression of PPARγ through increasing physical activity performance, UCP2 expression, and thereby mitochondrial metabolism decreases fat mass and obesity. It is possible that these gens have a synergistic effect and omega-3 fatty acids boost the effects of both. On the other hand, there are no studies that assess omega-3 fatty acids, PPARγ, UCP2, and their combined effects on blood lipid profiles and body fat percentage. Therefore, the results of this trial can be used as baseline information for conducting further clinical and sport nutrition studies.

## Conclusion

This study seeks to determine the effects of omega-3 fatty acids on PPARγ and UCP2 expressions, blood lipid profiles, and body composition. The results of this trial can be used as baseline information for conducting further clinical and sport nutrition studies.

## Strengths and limitations

This study addresses a piece of important evidence that show the effect of omega-3 supplementation on body metabolism-related genes; simultaneous measuring of the ucp2 gene as a central metabolic gene and ucp2 protein as a metabolism-related protein; assessment of the short-term effect of omega-3 supplementation on appetite and metabolic status; and double blind-RCT.

A limitation of this study is the differential effects of EPA and DHA were not assessed. Another limitation is that as PPARγ is a nuclear receptor, it requires a ligand for its activation and subsequent nuclear translocation. Compared to its expression levels, its protein level is very important, unfortunately, this study could not measure it. The evaluation of expression levels of downstream target genes other than UCP2 is suggested for future research. Also, we assess the physical activity just by one questionnaire (GPAQ) that could be very subjective.

## Data accessibility statement

The data collection forms are available in Persian from the corresponding author upon reasonable request. The data are not yet available.

## Abbreviations

UCP2: Uncoupling protein2

PPARγ: Peroxisome proliferator-activated receptor γ

MET: Metabolic equivalent of task

PBMC: Peripheral blood mononuclear cell

GWAS: Genome-wide association studies

EPA: Eicosapentaenoic acid

DHA: Docosahexaenoic acid

BMI: Body mass index

ATP: Adenosine triphosphate

GPAQ: Global Physical Activity Questionnaire

EDTA: Ethylene di amine tetra acetic acid
